# Best practices for standardized performance testing of infrared thermographs intended for fever screening

**DOI:** 10.1371/journal.pone.0203302

**Published:** 2018-09-19

**Authors:** Pejman Ghassemi, T. Joshua Pfefer, Jon P. Casamento, Rob Simpson, Quanzeng Wang

**Affiliations:** 1 Center for Devices and Radiological Health, U.S. Food and Drug Administration, Silver Spring, Maryland, United States of America; 2 Engineering Measurement Division, National Physical Laboratory, Teddington, United Kingdom; Institute of Materials Science, GERMANY

## Abstract

Infrared (IR) modalities represent the only currently viable mass fever screening approaches for outbreaks of infectious disease pandemics such as Ebola virus disease and severe acute respiratory syndrome. Non-contact IR thermometers (NCITs) and IR thermographs (IRTs) have been used for fever screening in public areas such as airports. While NCITs remain a more popular choice than IRTs, there has been increasing evidences in the literature that IRTs can provide great accuracy in estimating body temperature if qualified systems are used and appropriate procedures are consistently applied. In this study, we addressed the issue of IRT qualification by implementing and evaluating a battery of test methods for objective, quantitative assessment of IRT performance based on a recent international standard (IEC 80601-2-59). We tested two commercial IRTs to evaluate their stability and drift, image uniformity, minimum resolvable temperature difference, and radiometric temperature laboratory accuracy. Based on these tests, we illustrated how experimental and data processing procedures could affect results, and suggested methods for clarifying and optimizing test methods. Overall, the insights into thermograph standardization and acquisition methods provided by this study may improve the utility of IR thermography and aid in comparing IRT performance, thus improving the potential for producing high quality disease pandemic countermeasures.

## 1. Introduction

### 1.1. Infrared (IR) thermography medical applications

IR thermography—also known as thermal imaging—is a non-contact and noninvasive imaging approach that has been exploited for a wide range of biomedical and non-biomedical applications. It has been studied for use in cancer imaging [1–7], ischemic monitoring and vascular disease [8, 9], wound assessment [10], corneal temperature measurement [11–13], diagnosis of rheumatologic diseases [14], fever screening [15–26], *etc*. However, significant difficulties have been encountered in clinical translation of IR thermography. Standardization in different aspects of medical thermography, including tools (both acquisition hardware and processing algorithm) and examination environment, has always been a general agreement among the community of medical thermography [27]. The concept of standardization in thermography was introduced by the European Association of Thermography in 1978 and later a proposal for a standard was outlined by Clark *et al* [27]. Factors such as examination room conditions (temperature, humidity, *etc*.), thermographic imager accuracy, and image analysis methods were discussed in this proposal. Thereafter, with the steady increase in development and utility of IR thermography in medical applications, more detailed criteria for deployment of IR thermography were discussed and presented, resulting in improved diagnostic capability and reliability [28, 29]. Following two guidance documents published by the Standards, Productivity and Innovation Board of Singapore (SPRING Singapore), the International Standards Organization (ISO) produced two documents [18]: one describes the essential performance specification of suitable imaging systems for fever detection in humans [30], and the other describes the deployment, implementation and operational guidelines for identifying febrile humans using a screening thermograph [31]. These two documents lay a foundation for the best practices of evaluation and application of fever-screening IR thermographs (IRTs). Many parameters are defined in these documents. Assessment of these parameters is more important for absolute temperature measurement (*e*.*g*. fever screening) than relative temperature measurement.

IRTs (also known as IR/thermal cameras) and non-contact IR thermometers (NCITs) are the only currently viable temperature measurement approaches for mass screening of infectious disease pandemics, like the recent Ebola virus disease outbreak in West Africa [32], severe acute respiratory syndrome (SARS) outbreak in 2003 [33], and the influenza A pandemic (H1N1) outbreak in 2009 [34]. IRTs and NCITs enable nearly real-time estimation of body temperature by detecting IR emissions, which may result in more prompt quarantine of infectious individuals. Currently, NCITs remain a more popular choice for fever screening, and all IRTs cleared by the U.S. Food and Drug Administration have been limited to use in an adjunctive capacity and/or as a relative measurement. A document from the Centers for Disease Control and Prevention indicates that IRTs are not as accurate as NCITs and may be more difficult to use effectively [35]. However, there has been increasing evidences in the literature that IRTs can provide greater accuracy in estimating body temperature than NCITs. A study directly compared an NCIT to three IRTs indicated that the NCIT was less accurate [36]. Another study has shown that forehead skin temperatures measured by most NCITs are a much less reliable indicator of fever than inner canthi temperatures, the suggested measuring spots for IRTs [19, 30, 37].

As a result of the building evidences from IRT studies, technical organizations of the International Electrotechnical Commission (IEC) [30] and the European Association of Thermology [38], have concluded that IRTs offer an accurate method for fever screening if they have qualified performance and appropriate procedures are consistently applied. There have been standard and technical report recommending specific device performance testing requirements as well as best practices for thermographic fever screening [30, 31]. Currently, IEC 80601-2-59 is the only international standard for performance evaluation of IRTs intended for fever screening. However, some testing procedures and performance characteristics in this standard are not well-defined and the reference papers provide no discussion of their suitability or practical demonstration of their implementation. The purpose of this study is to evaluate, optimize and demonstrate the utility of a battery of test methods for standardized, objective and quantitative assessment of IRT performance based on IEC 80601-2-59. Preliminary results of this work have been presented in a conference [39].

### 1.2. Theory of IR thermography

Unlike most medical imaging approaches, IR thermography does not require irradiation, and thus presents no hazard to tissue. IR radiation emitted from biological tissues is detected and used to calculate temperature distributions. These calculations often account for radiation received from other sources such as the ambient and the emission of surroundings reflected by the object.

The IR emission from an object has a continuous spectrum of radiant energy, which varies with wavelength (λ) and its temperature (T, in kelvin); this can be described by a term known as spectral radiance (*L*_*b*_). The *L*_*b*_ distribution of a blackbody as a function of λ and T can be described by the Planck’s law as follows:

$$L_{b}(\lambda ,T)\;=\;\frac{2hc^{2}}{\lambda^{5}}{\left[\text{exp}\;\left(\frac{hc}{k\lambda T}\right)-1\right]}^{-1},\;W∙m^{-2}∙{\mu m}^{-1}$$ (1)

where h = 6.626176×10^−34^ J·s is the Planck’s constant, c = 299792458 m·s^-1^ is the speed of light in vacuum, and k = 1.380662 ×10^−23^ W·s·K^-1^ is the Boltzmann constant.

Integration of the area underneath the *L*_*b*_(*λ*, *T*) versus λ curve provides the total radiant energy of the blackbody (*E*_*b*_) when its temperature is T, expressed by the Stefan-Boltzmann formula as *E*_*b*_ = *σ*.*T*^4^. Usually, an object emits only a fraction of the radiant energy emitted by a blackbody at the same temperature. If the emissivity of the object is constant and independent of *λ*, the object is a graybody and its radiant energy can be expressed by the Stefan-Boltzmann formula for a graybody as follows [40]:

$$E_{g}\;=\;\varepsilon_{g}∙\tau_{atm}∙\sigma ∙T^{4},$$ (2)

where *E*_*g*_ [W·m^-2^] is the graybody’s radiant exitance, *ε*_*g*_ [–] denotes the graybody’s emissivity value (between 0 and 1), *τ*_*atm*_ [–] is the transmittance of the ambient (between 0 and 1), and *σ* is the Stefan-Boltzmann constant equal to 5.67×10^−8^ W·m^-2^·K^-4^ [41]. In general, *τ*_*atm*_ is estimated by knowing the object distance from the imager and the ambient relative humidity.

As the temperature of a blackbody increases, the intensity of its thermal radiation increases and the spectral distribution shifts towards shorter wavelengths. The peak wavelength (λ_max_) of the spectral distribution of a blackbody is described by Wien’s displacement Law:

$$\lambda max\;=\;\frac{b}{T}$$ (3)

where *b* is Wien’s displacement constant, equal to 2.898×10^−3^ m⋅K [42], derived from Plank’s constant and Boltzmann’s constant. The *λ*_*max*_ of a graybody can be approximated by the *λ*_*max*_ of a blackbody. For biological tissue in the physiological temperature range of 35°C to 41°C, the spectrum of emitted IR radiation peaks at about 9.3 μm, with the spectral regions of greatest radiation intensity extending from mid-wavelength IR (MWIR, 2.5–7 μm) to long-wavelength IR (LWIR, 7–15 μm) regions [43]. Thus, IRTs for clinical use often operate in MWIR or LWIR ranges.

## 2. Materials and methods

In this study, we have focused on performance evaluation of IRTs. Specifically, we have studied the recommended test methods for key characteristics: stability and drift, uniformity of the workable target plane, minimum resolvable temperature difference (MRTD), and radiometric temperature laboratory accuracy. [30] Furthermore, we have implemented optimized test methods to compare two commercial IRTs. It is our intent that accomplishing these goals will facilitate development of IRTs capable of effective fever screening. While we are also conducting an extensive human study on fever screening, such work is beyond the scope of this paper.

### 2.1. Standard specifications

The following is a summary of the terms and conditions described in the IEC 80601-2-59 standard [30] relevant to the performance evaluation test methods addressed in this paper. All the mentioned clauses (*e*.*g*., clause 201.101.6) in this paper are cited from this standard unless otherwise specified. A *screening thermograph* (ST) is composed of an IRT and an *external temperature reference source* (ETRS, usually a blackbody with known temperature and emissivity—clause 201.3.205), and in some cases, a computer and software for data acquisition, processing and storage. A *calibration source* (CS, a highly accurate blackbody with known and traceable temperature and emissivity—clause 201.3.202) is employed as a target to perform standard compliance tests. A *workable target plane* (WTP) is a specific region of target plane that meets the performance requirements (clause 201.3.215). Images of WTPs are used for temperature measurement. A minimum WTP image resolution of 320 (horizontal) × 240 (vertical) is required (clause 201.12.2.103). During each test, the temperature should be maintained between 18°C and 24°C and relative humidity between 10% and 75% (clause 201.5.3). Airflow from ventilation ducts should be deflected to minimize forced cooling or heating of the target (clause 201.7.9.3.9). The laboratory space chosen for IRT performance evaluation should be checked to ensure that no source of IR radiation (*e*.*g*., incandescent and halogen lightings) surrounds the experimental setup (clause 201.7.9.3.9).

### 2.2. Methodology and experimental setup

We used two commercial uncooled microbolometer IRTs sensitive to the LWIR band: IRT-1 (A325sc, FLIR Systems Inc., Nashua, NH) and IRT-2 (8640 P-series, Infrared Cameras Inc., Beaumont, TX). Nominal IRT specifications are listed in Table 1. The IRTs were attached to a stable platform and images were acquired with manufacturer-provided software. A hand-held weather meter, WM (Kestrel 4500NV, Weather Republic LLC, Downingtown, PA) was used to measure the ambient temperature and relative humidity, which are input parameters for the IRTs. The IRTs were stabilized for 15 minutes prior to each test (clause 201.101.8). A separate experiment was performed on each IRT to make sure that 15 minutes was long enough for stabilization. Measurement instability was considerably small during the normal operational condition (after the stabilization period), especially when used together with an ETRS (data are not shown here).

**Table 1:** Specifications of IRTs claimed by the manufacturers.

IRT	Spectral range [μm]	Operating range [°C]	NETD^a^ [mK]	Image sensor dimensions [pixels]	Field of view^b^: H,V [degrees]	Detector pitch [μm]	Dynamic range [bit]	Data streaming	Accuracy [°C]
IRT-1	7.5–13	-20 –+120	<50 @ 30°C	320 × 240	17, 14	25	14	Ethernet	±2
IRT-2	7–14	-40 –+500	<20 @ 30°C	640 × 512	30, 25	17	14	USB	±1

^a^ Noise equivalent temperature difference, used to grade camera thermal sensitivity.

^b^ Field of view is reported in horizontal (H) and vertical (V) directions.

Two extended area blackbodies with high temperature accuracy, stability, uniformity and emissivity, and low drift were used for IRT performance testing: BB-1 (SR-33N-4, CI Systems Inc., Simi Valley, CA) and BB-2 (SR-800R-4D, CI Systems Inc.). The blackbodies had the same emitter size of 4×4 inch^2^. BB-1 was used as an ETRS and BB-2 as a CS. Technical specifications of the blackbodies are listed in Table 2. All data in Table 2 were claimed by the manufacturer without independent calibration except for the total system uncertainty values that were calculated based on the claimed data. Each blackbody can be set to a target temperature within its operating range. BB-1 has an embedded controller and works in absolute mode. It is claimed to be highly accurate, with total system uncertainty of *u*_*BB*1_ = ±0.04°C or expanded uncertainty of *U*_*BB*1_ = *k* · *u*_*BB*1_ = ±0.08°C (*k* = 2 is the coverage factor for a confidence interval of approximately 95% [44]) and combined stability and drift of ±0.02°C, which satisfies the standard requirements (maximum expanded uncertainty of ±0.3°C, maximum combined stability and drift of ±0.1°C, clause 201.101.3.1). BB-2 was claimed to have superior accuracy and stability compared to BB-1. BB-2 was used as a CS for characterizing the STs and can be operated in absolute or differential modes. The expanded uncertainty (*U*_*BB*2_ = ±0.08°C) and combined stability and drift (±0.02°C) of BB-2 were the same as those of BB-1, which meets the standard requirement (maximum expanded uncertainty of ±0.2°C, maximum combined stability and drift of ±0.05°C, Annex BB in [30]). Both blackbodies were claimed to be traceable to the NIST ITS-90 thermocouples database.

**Table 2:** Specifications of blackbodies claimed by the manufacturer (under normal lab environment,18–24°C)^a^.

Blackbody	Mode	Operating range [°C]	Aperture size [inche^2^]	Uniformity ^b^ [°C]	Accuracy [°C]	Stability [°C]	Yearly Drift [°C]	Total System Uncertainty ^c^ [°C]	Emissivity [–]
BB-1	Absolute	5–100	4×4	0.01	0.05	0.01	0.02	±0.04	0.98±0.01
BB-2	Absolute	0–125	4×4	0.01	0.007	0.008	0.02	±0.04	0.98±0.01
	Differential	-25–100			0.008				

^a^ The data for uniformity, accuracy, stability and drift were based on worst-case scenarios.

^b^ Uniformity values were for a ±1°C step from ambient temperature at 80% of the central area. For other temperature, multiply by ΔT (the difference between the ambient and the blackbody temperatures).

^c^ Calculated based on the standard uncertainty value of uniformity, stability and drift. Assuming ambient temperature of 20°C and blackbody temperature of 35°C.

The standard [30] recommends use of a CS with emissivity ≥ 0.998 (Annex BB). The nominal emissivity of BB-1 and BB-2 was 0.98±0.01. We are not aware of any available commercial blackbody that meets the standard’s requirements in the intended minimum temperature imaging range of 30°C to 40°C (clause 201.101.2.1). We did not find the justification for the specified emissivity in the standard. The references in the standard do not mention the emissivity of 0.998. While some cavity blackbodies have emissivity around 0.99, they usually operate at temperature higher than 50°C.

Consequently, to ensure an accurate temperature estimate, recorded thermograms were compensated for non-ideal blackbody emissivity, *ε*_*g*_, (*e*.*g*., non-zero reflectivity) per the Stefan-Boltzmann formula for a graybody. Thus, IR emission of an object can be expressed as [40]:

$$E_{total}\;=\;\varepsilon_{g}∙\tau_{atm}∙\sigma ∙T^{4}+(1-\varepsilon_{g})∙\tau_{atm}∙\sigma ∙T_{refl}^{4}\;,$$ (4)

where *E*_*total*_ [W·m^-2^] is the total radiosity received by the camera, *ε*_*g*_ is considered as 0.98 for our blackbodies, (1 − *ε*_*g*_) denotes the object’s reflectivity, and *T*_*refl*_ [K] represents the reflected temperature. The reflected temperature could be measured based on the “reflector” method specified in a standard [45]. A thermographic inspection of the laboratory (*e*.*g*., walls, air vents, and devices) was conducted using a hand-held IRT (FLIR ONE, FLIR Systems Inc., Nashua, NH). Since no external source of IR radiation was found, the reflected temperature was assumed to be the same as the ambient temperature. The difference between the temperatures calculated based on measured *T*_*refl*_ and assumed *T*_*refl*_ over the range from 30°C to 40°C is less than 0.08%, confirming that the assumption is reasonable. Approximating *T*_*refl*_ with the ambient temperature is for ease of testing. However, this approximation should be verified for a given system under a given environment. In case of the presence of an excessive surrounding IR radiation, reflected temperature should be measured per ASTM recommendations [45]. Ambient IR transmissivity could be less than unity in long distance imaging and high relative humidity conditions. But given the current testing at 0.8 m, an assumption of *τ*_*atm*_ = 1.0 was implemented. The measuring distance, relative humidity and ambient temperature can affect *τ*_*atm*_ and such assumption should be verified for a different system under a different environment.

**Fig 1:**
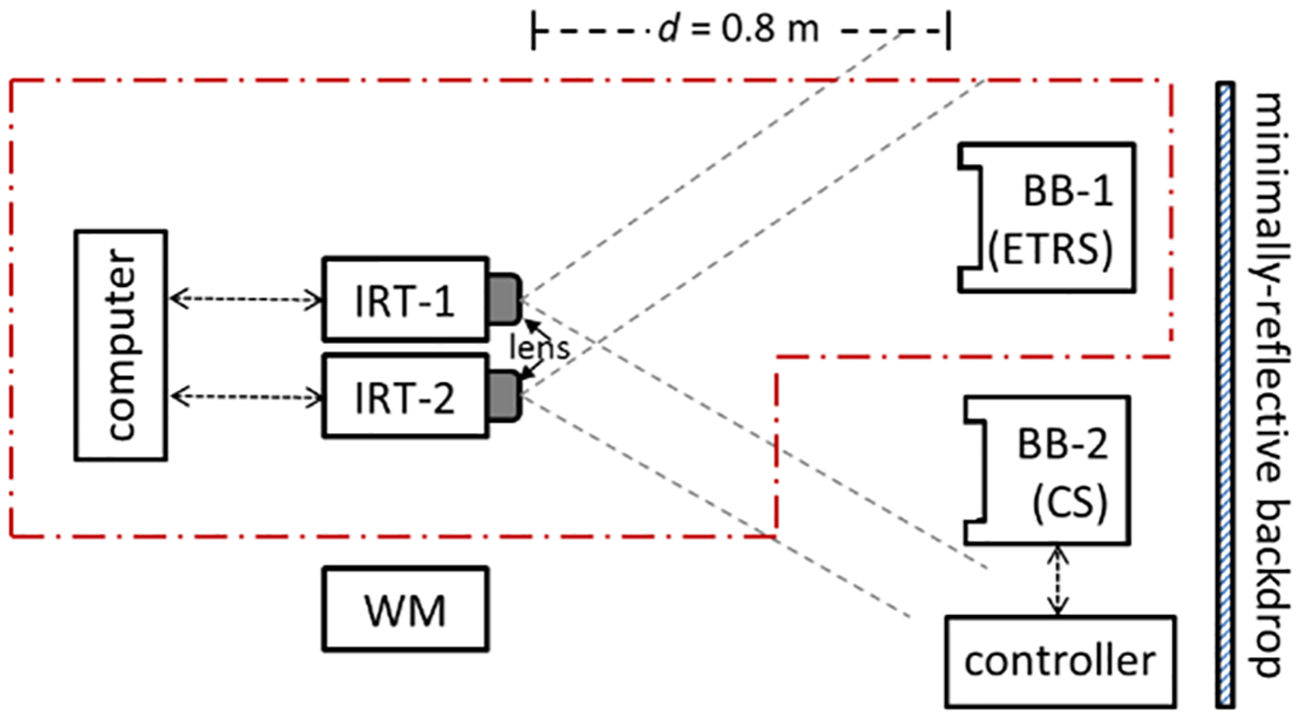
Block diagram of the experimental setup. (Red dotted box shows the screening thermograph system. Working distance was d = 0.8 m.).

A block diagram of the experimental setup is shown in Fig 1. The blackbodies were positioned in front of the IRTs, 1.5 m above the floor, in an in-focus plane (exception: BB-2 was out of focus during the uniformity tests at short working distance), and perpendicular to the line of sight of the imagers (clauses 201.3.213 and 201.101.7). The working distance between the blackbodies and the IRTs was *d* = 0.8 m (exception: working distance was set to 0.15 m for IRT-1 and 0.05 m for IRT-2 for short distance uniformity test and 0.35 m for MRTD evaluation inside a temperature chamber, respectively). For normal fever screening, the working distance should ensure thermograms with minimum dimensions of 240×180 pixels for the subject’s face (clause 201.12.2.103) and 20×20 pixels for the ETRS (clause 201.101.3.2). Given the sensor dimensions and field of view (FOV) of IRT-1 and IRT-2, as listed in Table 1, a distance of 0.8 m is needed to achieve a 240×180 pixels thermogram of the face with a spatial resolution (clause 201.101.9) of ~1 mm /pixel (*i*.*e*., one pixel can image an area of 1×1mm^2^ on the face). The working distances for IRTs with different sensor pixel numbers and camera FOV might be different. The size of ETRS active area is recommended to be less than 10% of the face during fever screening (Annex AA); however, we have shown that a larger size (15–20%) is also acceptable if it can be experimentally proven that the ETRS doesn’t adversely affect the measurement. A cloth backdrop with low reflectivity was used to minimize reflected IR radiation from the surroundings (clause 201.7.9.3.9). The emissivity of the cloth was measured based on the “noncontact thermometer” method [46]: *ε*_*g*_ = 0.91±0.02 (experimental details not provided here).

### 2.3. Performance test methods

As shown in Fig 1, the main devices used in this study include IRT-1, IRT-2, BB-1, BB-2, and WM. A computer with controlling software is considered part of an IRT. A screening thermograph (ST) usually includes an IRT and a blackbody as an ETRS (BB-1 in this case) for temperature offset compensation to ensure precise operation between calibrations (clause 201.3.209). If the IRT alone satisfies the standard level of performance (stability and drift, and laboratory accuracy), the ETRS could be avoided. In this study, we checked the performance of two STs: ST-1 that includes IRT-1 and BB-1, and ST-2 that includes IRT-2 and BB-1. The effect of the ETRS on the results was also investigated.

#### 2.3.1. Stability and drift

To estimate the system stability, the test procedure described in clause 201.101.4 was followed. The CS (BB-2 set at 37°C) was placed in the WTP of the ST-1 (and ST-2) and totally 1,920 consecutive frames were captured for 8 hours with time steps of 15 seconds (any time steps between 5–15 seconds is acceptable according to the standard). For evaluation of ST-1 and ST-2, captured BB-2 frames were corrected for temperature offset error using the ETRS (BB-1). A region of each frame within the BB-2 aperture—in this case a 60×60 pixels square excluding BB-2’s four edges to avoid data inaccuracy—was extracted for calculations. Then the mean temperature value within the extracted region, *M*_*frame*_, was calculated for each frame. Afterwards, the mean and standard deviation (SD) of the 1,920 *M*_*frame*_ values for 8 hours of measurement (*M*_*8h*_ and *SD*_*8h*_) were calculated. The standard requires that three times *SD*_*8h*_ –the confidence interval of the observations that is greater than 99% [44]–should be less than 0.1°C. However, the standard doesn’t define the standard uncertainty and expanded uncertainty of stability (*u*_*S*_ and *U*_*S*_, where *u* means standard uncertainty, *U* means expanded uncertainty, and the subscript *s* means stability). We recommend *u*_*S*_ be defined as *SD*_*8h*_. Then 3·*SD*_*8h*_ is expanded uncertainty (*U*_*S*_) with coverage factor k = 3 and confidence level >99%. The value of *u*_*S*_ is used to calculate the standard uncertainty of the ST (Eq 8) and should be less than 0.03°C (0.1/k = 0.03).

For drift analysis, the stability evaluation procedures should be repeated every day for the device’s calibration interval or two weeks, whichever is longer. The requirement for drift is that the maximum difference between *M*_*8h*_ values (*M*_*8h*,*max*_*—M*_*8h*,*min*_) should be less than 0.1°C. The standard doesn’t explain how to translate the results into standard uncertainty of drift (*u*_*d*_). Unlike the random property of stability, drift data can change monotonically with time, *i*.*e*., measured temperature keeps drifting away from the true values. Therefore, we define *u*_*d*_ as (*M*_*8h*,*max*_*—M*_*8h*,*min*_) based on the worst-case scenario.

Since all the IRTs and blackbodies in this study have a one-year manufacturer-recommended calibration interval, the calibration interval for ST-1 and ST-2 can also be considered as one year, assuming the data from the manufacturers are reliable. According to the standard, a one-year measurement should be performed to accomplish a thorough drift analysis for the systems. Since the ETRS has high stability, small drift, and high spatial uniformity (Table 2) and the goal of this paper is to optimize test methods instead of evaluating devices, we only evaluated drift over a two-week period to demonstrate the evaluation process. Furthermore, stability and drift values of IRT-1 and IRT-2 (*i*.*e*., no offset compensation by the ETRS) were also measured and results were compared to the values of ST-1 and ST-2.

Per the standard, both stability (3·*SD*_*8h*_) and drift (*M*_*8h*,*max*_*—M*_*8h*,*min*_) values should be less than 0.1°C, and the combined stability and drift less than 0.2°C (clause 201.101.4). The requirements are not clear from the statistics point of view. They had better be based on standard uncertainty, combined standard uncertainty, or expanded uncertainty due to stability and drift. [47] A better expression of the requirements for stability and drift is that *u*_*S*_ should be less than 0.03 °C and *u*_*d*_ should be less than 0.1°C. The criterion for combined stability and drift is redundant and can be removed.

#### 2.3.2. Uniformity of WTP

Uniformity or spatial variation across the WTP is an important performance factor for a focal plane array [48, 49]. To investigate the utility of the recommended method for determining an ST image uniformity (clause 201.101.6), the BB-2 blackbody with superior temperature uniformity (Table 2), was maintained at 37°C (It can be set at other temperatures in the 34°C to 39°C range) and at the 0.8 m working distance from the imager lens (Fig 1). BB-2 was repositioned at 29 locations throughout the WTP including four corners, center, and 24 random locations. At each location, a thermal image of BB-2 was captured by the ST. BB-2 temperature was recorded from a single image pixel value (no averaging algorithm was implemented) of the thermal image. Uniformity of the WTP was then calculated from the maximum difference between the 29 measured values. The uniformity value of an ST should be less than 0.2°C (clause 201.101.6). A larger uniformity value indicates worse uniformity. This procedure was performed separately for ST-1 and ST-2. For this study, this method is called IEC-29-pixels method.

The IEC-29-pixels method is time consuming and vulnerable to artifacts associated with the uniformity and repeated repositioning (*e*.*g*., changes in target-IRT distance and relative angle) of BB-2. A modified-29-pixels method was performed by placing the extended area BB-2 close to the IRT lens so that the blackbody aperture can cover the entire FOV of the IRT. In this study, BB-2 was placed 0.15 m from the IRT-1 lens and 0.05 m away from the IRT-2 lens. A single image was then captured to assess the uniformity based on 29 pixels similar to the IEC-29-pixels method (clause 201.101.6), *i*.*e*., four corners, center, and 24 random pixels.

In the modified-29-pixels method, all pixels of the captured IR image can be used to provide a comprehensive uniformity map of the entire FOV. This may also be beneficial for finding an optimal WTP (with best uniformity) throughout the entire FOV for an IRT with a sensor exceeding the required image dimensions of 320×240 pixels. Since all pixel intensities are known from the short distance image, a more comprehensive uniformity analysis could be performed by looking at the intensities of all pixels rather than only 29 pixels—referred to as all-pixels method in this paper.

The IEC-29-pixels, modified-29-pixels and all-pixels methods are all based on the largest intensity difference between pixels. While the standard uncertainty of uniformity (*u*_*U*_) should be used to calculate the radiometric temperature laboratory accuracy (Eq 8), it is difficult to translate the uniformity results based on the IEC-29-pixels and modified-29-pixels methods to *u*_*U*_ values since it is difficult to define the distribution function of the limited 29 data. As shown later, the total number of sampled pixels will significantly affect the results and the repeatability of these method is not good. On the other hand, intensity distribution of all the pixels from a uniform blackbody can be evaluated with SD and the SD of all the pixel can be directly considered as *u*_*U*_. We call the method based on the SD of all pixels as the all-pixels-SD method.

The effects of IRT focal length, BB-2 focus state (*i*.*e*., whether BB-2 is in focus), and BB-2 temperature on the uniformity results were also studied. We evaluated two focal lengths by focusing the IRTs at 0.8 m and 0.15 m from IRT-1 lens and 0.8 m and 0.05 m from IRT-2 lens. For a given focal length, BB-2 was either in focus or out of focus at these two distances. The effect of BB-2 temperature was studied at 31, 33, 35, 37 and 39°C.

#### 2.3.3. Minimum resolvable temperature difference (MRTD)

The MRTD reveals the smallest temperature difference that an IRT can consistently detect within its WTP. It determines the IRT efficiency for discriminating details in a scene and provides insights into its sensitivity and spatial resolution. In this study, MRTD compliance was checked based on the ASTM E1213-14 standard [50]. The MRTD test target (named 4-bar target) was an aluminum plate with high-emissivity coating. Five groups of four bars were etched away to obtain five spatial frequencies, between 0.04 to 0.2 cycles/mrad (measured at the working distance of 0.8 m). The 4-bar target was mounted in front of the extended surface of BB-2 so the BB-2 surface can be seen through the etched bars. The aspect ratio of each bar was 1:7. The differential temperature (ΔT) between the etched bars (the background BB-2) and the conjugate bars (the area on the aluminum plate between the etched bars, maintained at the ambient temperature) was adjusted using the BB-2 controller. IRTs were focused on the target, located in the center of the WTP with the bars in the vertical direction, and images were captured and viewed on a monitor with proper brightness and contrast. The resolution of the monitor should be high enough so that the MRTD is only affected by the input thermal images instead of the monitor. Initially, ΔT was set to zero, then gradually increased in increments of 0.005°C, until the observer could visually distinguish the four bars on the screen. The mean temperatures of the etched bars and the conjugate bars were then calculated from captured thermograms, and their difference was recorded. No offset compensation is necessary for this experiment since the etched and conjugate bars are imaged in the same frame at the same time. The test was then repeated with three other observers. For each particular spatial frequency, the lowest recorded temperature difference with a detection probability of at least 50% was selected as the MRTD value [50] (in this study at least two of the four observers shall resolve the bars). The data were then plotted against spatial frequency as a metric of IRT performance. The MRTD shall be less than or equal to 0.1°C (clause 201.101.5).

In addition to spatial frequency, MRTD of an IRT can also be affected by the target/ambient temperature [48], particularly in situations where the imager response characteristic curve is non-linear. For further investigation, a similar experimental setup was placed inside a controlled temperature chamber (Hotpack, Warminster, PA). Due to the chamber size constraint, working distance was decreased from 0.8 m to 0.35 m. Therefore, a different range of spatial frequencies was achieved with the same 4-bar target: from 0.018 to 0.088 cycles/mrad. MRTD values were measured at five target temperatures between 31°C and 39°C (with 2°C increments) with the same procedure described before. The direction of the bars can also affect temperature sensitivity and spatial resolution due to the combination of the data transfer architecture and the pixel design. Therefore, MRTD values were measured with the 4-bar target in both vertical and horizontal directions.

The aforementioned MRTD test method is subjective, and thus susceptible to reader variability which is not ideal for standardization. Objective MRTD test methods based on image analysis (*e*.*g*., contrast- or SNR-based evaluation) [51, 52] methods have shown promise, however a suitable threshold for MRTD estimation has not been identified. We demonstrated a contrast-based analysis approach using both IRTs with the conjugate bars on the target aligned horizontally and maintained at 35°C ambient temperature. Since the integration period of the human eye/brain system is about 0.2 seconds [53], thermograms of the test target were continuously captured for 0.2 seconds at a frame rate of 30 Hz resulting in 6 frames each for various known differential temperatures, ΔT (13 set points between 0°C to 0.1°C). Averaged thermograms were then generated from each image series. Contrast levels of the 4-bar target in the averaged thermograms at various ΔT values were measured. The contrast [–] can be expressed as:

$$contrast\;=\;\frac{\left|I_{etched}-I_{conj}\right|}{I_{etched}+I_{conj}}\;\times 100\%,$$ (5)

where *I*_*etched*_ and *I*_*conj*_ are the mean intensities of the etched bars and the conjugate bars after background intensity subtraction, respectively. Background intensity was measured from a 20×20 pixels square region of the target plate away from the bars. By defining a contrast threshold at which the bars are considered resolvable, MRTD values could be estimated from the corresponding ΔT.

#### 2.3.4. Radiometric temperature laboratory accuracy

For radiometric temperature laboratory accuracy testing, CS (BB-2) was placed in the WTP of each ST at the working distance (*d* = 0.8 m). The CS temperature was measured five times (clause 201.101.2.2) at each of the 11 set points (*T*_*CS*_) in increments of 1°C across the range of 30°C to 40°C. The minimum number of set points can be 5 (clause 201.101.2.2). The mean temperature value (*T*_*ST*_) at each *T*_*CS*_ setting was recorded from a region of interest described in Section 2.3.1 and corrected for temperature offset using ETRS (BB-1) data. The laboratory accuracy of the screening thermographs was evaluated based on the following criterion (clause 201.101.2.2):

$$|T_{ST}-T_{CS}|+|u|\leq 0.5\;,$$ (6)

where *u* is the combined standard uncertainty of the laboratory accuracy that can be determined as:

$$u^{2}\;=\;u_{CS}^{2}+u_{ST}^{2},$$ (7)

where *u*_*CS*_ is the standard uncertainty of the CS and *u*_*ST*_ is the standard uncertainty of the ST. Assuming the standard uncertainties due to drift (*u*_*D*_), stability (*u*_*S*_), uniformity of WTP (*u*_*U*_), MRTD (*u*_*MRTD*_), and ETRS (*u*_*ETRS*_) are independent and random, *u*_*ST*_ can be calculated as:

$$u_{ST}\;=\;\sqrt{u_{D}^{2}+u_{S}^{2}+u_{U}^{2}+u_{MRTD}^{2}+u_{ETRS}^{2}},$$ (8)

The values of *u*_*D*_, *u*_*S*_, and *u*_*U*_ were measured with the test methods in Sections 2.3.1 and 2.3.2. The value of *u*_*MRTD*_ was calculated based on the method in Section 3.3. The values of *u*_*CS*_ and *u*_*ETRS*_ were based on the manufacturer specifications (Table 2). The values of |*T*_*ST*_ − *T*_*CS*_| should comply with Eq 6 at every CS temperature point (clause 201.101.2.2).

## 3. Results

If not specified, the evaluation results in this section only came from the WTP regions of each IRT or ST. The WTP of IRT-1/ST-1 was the whole FOV and the WTP of IRT-2/ST-2 was only part of the FOV where the uniformity was the best (Fig 3). If the WTP regions are defined in a different way, the evaluation results might be different.

### 3.1. Stability and drift

**Fig 2:**
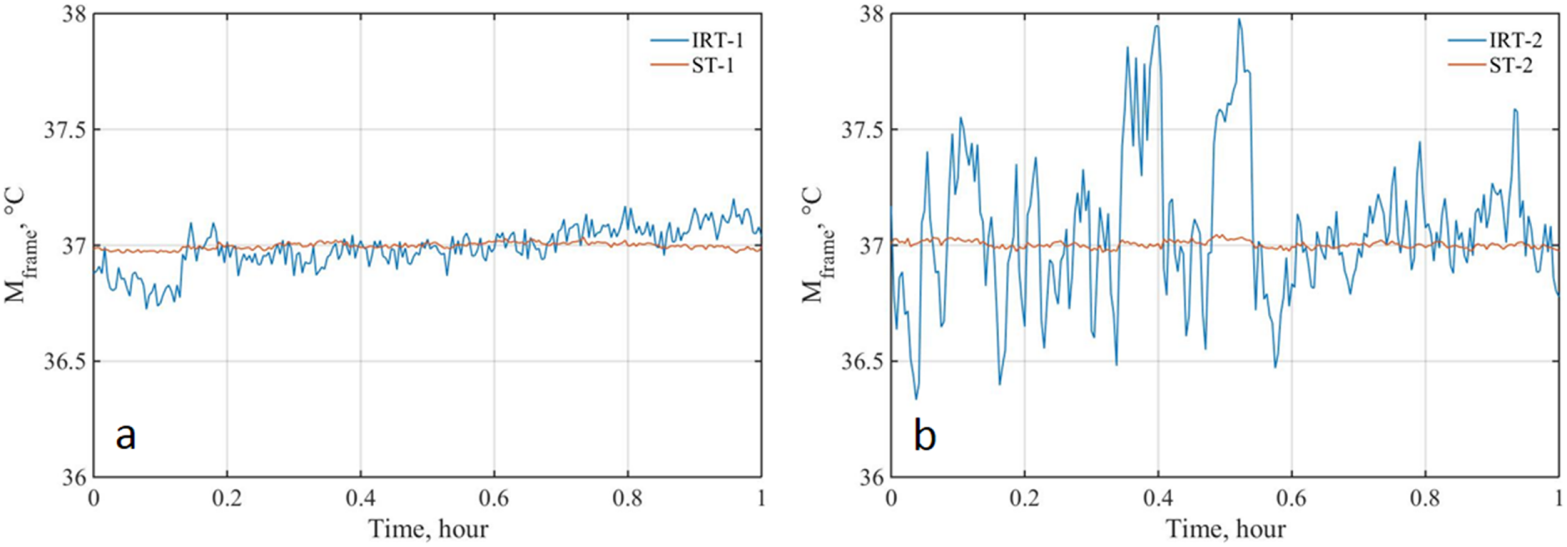
Stability results based on one-hour continuous temperature recordings with time interval of 15 seconds. (a: ST-1 and IRT-1; b: ST-2 and IRT-2).

One hour of a sample series of temperature data captured every 15 seconds is shown in Fig 2. Each data point represents the average blackbody temperature (*M*_*frame*_) from one frame. Data before (IRT-1 and IRT-2) and after (ST-1 and ST-2) offset compensation based on the ETRS are compared. The SDs of the *M*_*frame*_ values over an 8 hours period (*SD*_*8h*_, *i*.*e*., the standard uncertainty of stability *u*_*S*_) for IRT-1 and IRT-2 were 0.10°C and 0.27°C, respectively. These values were 0.02°C for ST-1 and ST-2. Results indicated that ST-1 and ST-2 showed comparable stabilities, however IRT-1 showed much lower temporal variation than IRT-2. The stability values of 3*SD*_*8h*_ were 0.06°C for ST-1 and ST-2 and both systems met the standard requirement (3*SD*_*8h*_ is less than 0.1°C—clause 201.101.4). However, when no ETRS is used, IRT-1 was more stable and both IRTs failed to satisfy the standard requirement. Temperature drift was less significant in ST-1 compared to ST-2, with the *u*_*d*_ values being 0.03°C and 0.08°C respectively. Both STs satisfied the standard requirements for drift (less than ±0.1°C—clause 201.101.4). When no ETRS was used (*i*.*e*., IRT-1 and IRT-2), neither drift nor stability values of the two devices satisfied the requirements.

To access the effect of sampling interval on stability and drift measurements, further analysis was performed. Slower data acquisition rates—capturing one frame every 30, 60, 120, and 300 seconds–were employed and stability and drift values were calculated (Table 3). Different sampling intervals showed similar stability and drift results. It appeared that a slower sampling rate of one frame per 300 seconds might be sufficient for stability and drift evaluation.

**Table 3:** Stability and drift values for different sampling intervals.

	ST	Sampling intervals, seconds				
		15	30	60	120	300
Stability, °C	ST-1	0.05	0.05	0.05	0.05	0.06
	ST-2	0.05	0.06	0.05	0.05	0.05
Drift, °C	ST-1	0.03	0.03	0.03	0.03	0.03
	ST-2	0.08	0.08	0.08	0.08	0.09

### 3.2. Uniformity

An area of 320×240 pixels (minimum requirement, can be larger) that has the best uniformity within the whole FOV was selected as the WTP of an ST by scanning the FOV with a moving window. For IRT-1, the whole FOV is the WTP since the sensor only have 320×240 pixels. The standard [30] required at least 29 points to evaluate the uniformity. Since the evaluation process is random, the uniformity results also show random behavior. We repeated the IEC-29-pixels method on a frame for 100 times and got the uniformity values ranged from 0.21 to 0.47 °C for IRT-1 and 0.24 to 0.56°C for IRT-2. Therefore, each uniformity value throughout this section is the mean from 100 repeated sampling process and is expressed with average and SD values. When BB-2 was set at 37°C, uniformity values within the WTPs were measured to be 0.32±0.05°C and 0.36±0.06°C for ST-1 and ST-2, respectively based on the IEC-29-pixels method. Neither of these results satisfies the standard requirement (less than 0.2°C—clause 201.101.6).

The modified-29-pixels method is less burdensome and more robust to artifacts associated with the uniformity than the IEC-29-pixels approach since it only needs one single frame and avoids repeated repositioning. Theoretically, for a fixed IRT focal length, the uniformity values by placing BB-2 at different distances (in-focus and out-of-focus situations) [49] should be rather close if BB-2 is highly uniform. The uniformity results with the IEC-29-pixels and modified-29-pixels methods should be close if the same focal length is used and no measurement artifacts are involved.

**Fig 3:**
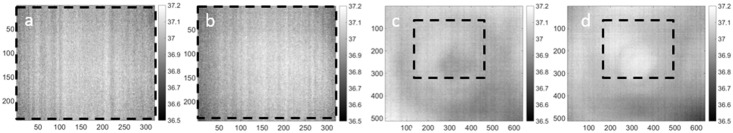
Sample thermograms of BB-2 fixed at 37°C, and placed at 0.15 m from IRT-1 (a and b) and 0.05 m from IRT-2 (c and d) lenses. (a and c: focused on BB-2; b and d: focused at 0.8 m; dotted box illustrates the WTP region in each image; the x and y coordinates show the pixel numbers in x and y directions).

The camera focal length might affect the uniformity results. Fig 3 displays thermograms of BB-2 (at 37°C) captured with IRT-1 and IRT-2 adjusted at two focal planes: 1) 0.15 m from IRT-1 or 0.05 m from IRT-2 (*i*.*e*., focused at the BB-2 location), and 2) 0.8 m from IRT-1 and IRT-2 (*i*.*e*., focused at the working distance). Two dominant spatial non-uniformities were recognized in IRT-1 at both focal lengths. The first is a vertical striping pattern likely generated during digitization or other post-processing procedures. This is not an artifact of BB-2 since the pattern remained unchanged after rotating the IRT (data not shown here). The second is a vignetting artifact (intensity reduction near the corners), likely caused by the lens. In IRT-2, striping artifacts are less apparent than in IRT-1. However, a dark circle when the camera focused at 0.05 m (and a brightened circle when the camera focused at the working distance) is seen in the center of the image, likely a narcissus effect due to IRT-2 optics [49]. A vignetting artifact, especially when the camera focused at the working distance, is also apparent in the IRT-2 image. These thermograms show the uniformity changes at different focal lengths for both IRT-1 and IRT-2 and the change for IRT-2 is more significant. Therefore, the camera should be focused at the working distance for uniformity evaluation to avoid the effect of focal length.

**Fig 4:**
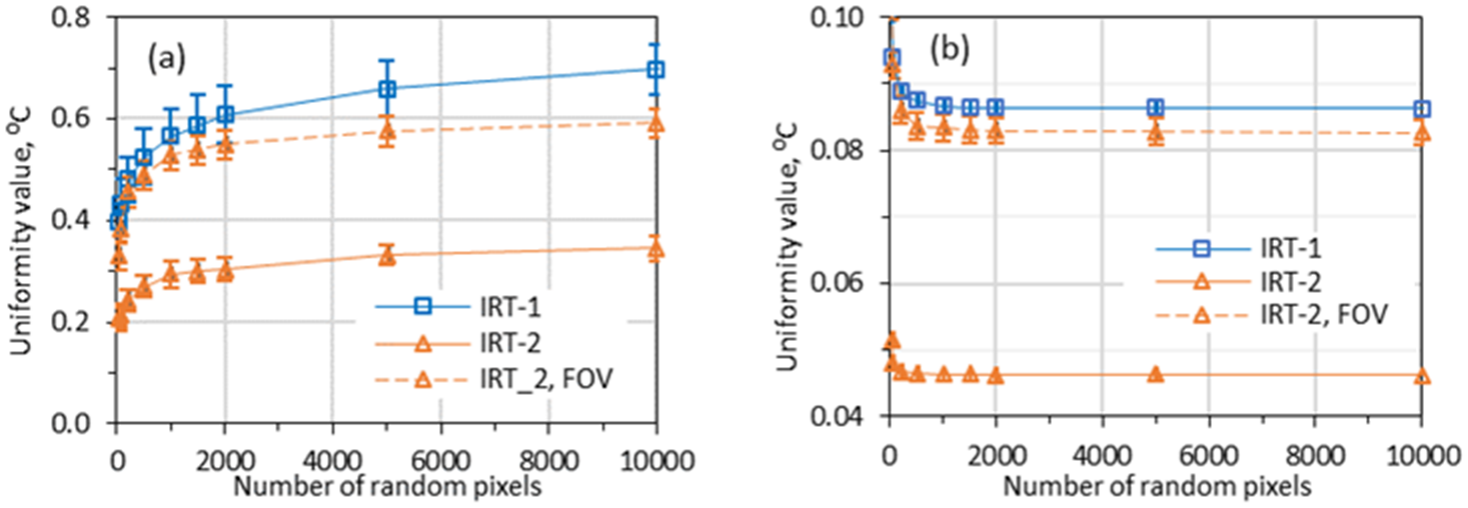
WTP and FOV uniformity values based on different numbers of random pixels. (a: maximum difference as uniformity; b: SD as uniformity; cameras focused at 0.8 m; BB-2 at 37 °C; error bars show SD of repeated sampling).

The total number of points for the evaluation also affect the results. Similar with the modified-29-pixels method, we selected different numbers of random pixels (including the center, four corners, and other randomly selected pixels) and calculated their maximum difference as the uniformity measure. For each number of random pixels, the random sampling process was repeated 100 times on a same frame and their average and SD (error bars) values were compared in Fig 4a. The results show that larger pixel number generally resulted in larger uniformity value (*i*.*e*., worse uniformity). Fig 4a shows that the requirement of at least 29 random pixels in the standard [30] does not guarantee a reliable uniformity value.

On the other hand, SD is often used as a measure for uniformity evaluation [43, 54]. Following a similar procedure for obtaining Fig 4a, we obtained Fig 4b) by using SD values as the measure for uniformity instead of using maximum difference. From Fig 4b, the SD values are stable with the change in pixel numbers, especially when the pixel number is larger than 1000. The figure also shows that for the same image, the SD values can distinguish the uniformity difference from different regions: the uniformity within the FOV (the “IRT-2, FOV” curve) is worse than the selected WTP (the “IRT-2” curve). The smaller error bars in Fig 4b than those in Fig 4a indicate a better repeatability of the SD measure than the maximum difference measure. Therefore, the all-pixels-SD method might be an alternative method for the IEC-29-pixels, modified-29-pixels, and all-pixels methods that are based on the largest intensity difference.

**Fig 5:**
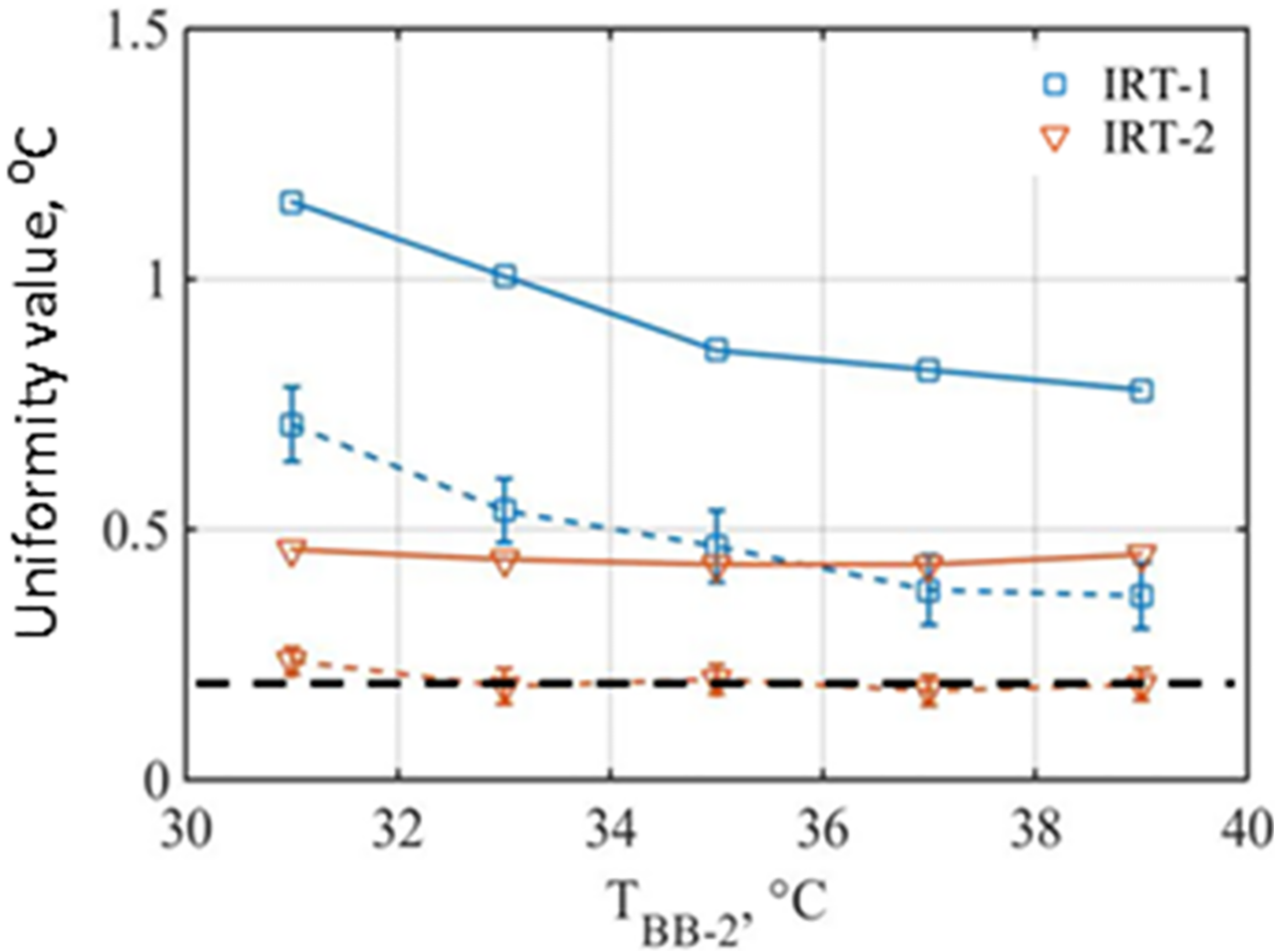
IRT uniformity values versus BB-2 temperature based on the modified-29-pixels (dashed lines), and all-pixels (solid lines) methods. (Dashed horizontal line at 0.2°C shows the IEC requirement. Cameras were focused at 0.8m).

The BB-2 temperature might also affect the uniformity results. A summary of uniformity measurements at different BB-2 temperatures is provided in Fig 5. In general, the uniformity values tended to be lower at higher temperatures for IRT-1 whereas the uniformity values are rather stable at different target temperatures for IRT-2. On average, IRT-2 showed a better level of uniformity than IRT-1 within the WTP per the modified-29-pixels and all-pixels methods. While IRT-2 benefits from its higher sensor resolution and larger FOV compared to IRT-1, the area outside of its WTP might not be as useful. The all-pixel method always resulted in higher level of non-uniformity as discussed in the previous paragraph. From Fig 5, IRT-1 failed to satisfy the IEC requirement regardless of the test method used. However, the uniformity values of IRT-2 across its WTP are around 0.2°C based on the modified-29-pixels method, showing an acceptable level of uniformity.

The uniformity can be improved by image processing algorithms in the IRT image processing pipeline. Fixed pattern noise can be removed with the help of a dark frame (for dark signal noise) or offset and gain for each pixel (for photon response noise). Temporal noise can be suppressed by averaging multiple images. Larger number of frames for averaging may lead to a higher level of uniformity for a static object, but require longer image acquisition time and may introduce blurring of the averaged image for a moving object. Presumably, motion artifact is negligible in standardized fever screening where subjects are asked to remain still at a given distance from the imager. While such image processing algorithms can be implanted in the IRT image processing pipeline to reduce image noise and improve uniformity before the output, they are not the topics of this manuscript. Our focus in this paper is on the evaluation of a whole thermograph system including its image processing pipeline (*i*.*e*., we consider the whole system including hardware and software as a black box). We consider images from the system as the final ones and should not be further processed to artificially improve the uniformity results.

The all-pixels method is a more comprehensive way of uniformity evaluation since it considers the entire pixels of WTP and is as fast as the modified-29-pixels approach. Moreover, finding a WTP with best uniformity within a FOV is easy using the all-pixels method. However, the uniformity value based on the all-pixels method is much larger than other methods since the value is calculated from the maximum and minimum values of all the pixels. On the other hand, the uniformity value will be rather stable if we use SD as a uniformity measure. Our data (Fig 4b) have shown that the SD values from randomly selected pixels are rather constant if the pixel number is larger than 1000. Therefore, a simple way of evaluating uniformity is to calculate the SD values from all the pixels within the region of interest. Of course, the uniformity criterion based on SD is different from the IEC-29-pexels criterion of 0.2°C. IRT-2 has uniformity values of ~0.2°C based on the modified-29-pixels method (Fig 5) and ~0.05°C based on the all-pixels-SD method (Fig 4b). Therefore, it is reasonable to mirror the IEC uniformity criterion of 0.2°C to a criterion values of approximate 0.05°C based on the all-pixels-SD method. The maximum difference and SD of a large number of pixel intensity values do have correlation. Statistically, if we assume the intensity values of the pixels has a normal distribution and the largest intensity difference of 0.2 °C cover 95.45% of confidence level (a confidence level covered by 4 times of SD values), then the SD of the pixel intensity values can be directly calculated as 0.05°C (0.2/4 = 0.05). Therefore, we propose to use the all-pixels-SD method to evaluate the uniformity and set the uniformity criterion as SD of 0.05°C.

### 3.3. MRTD

**Fig 6:**
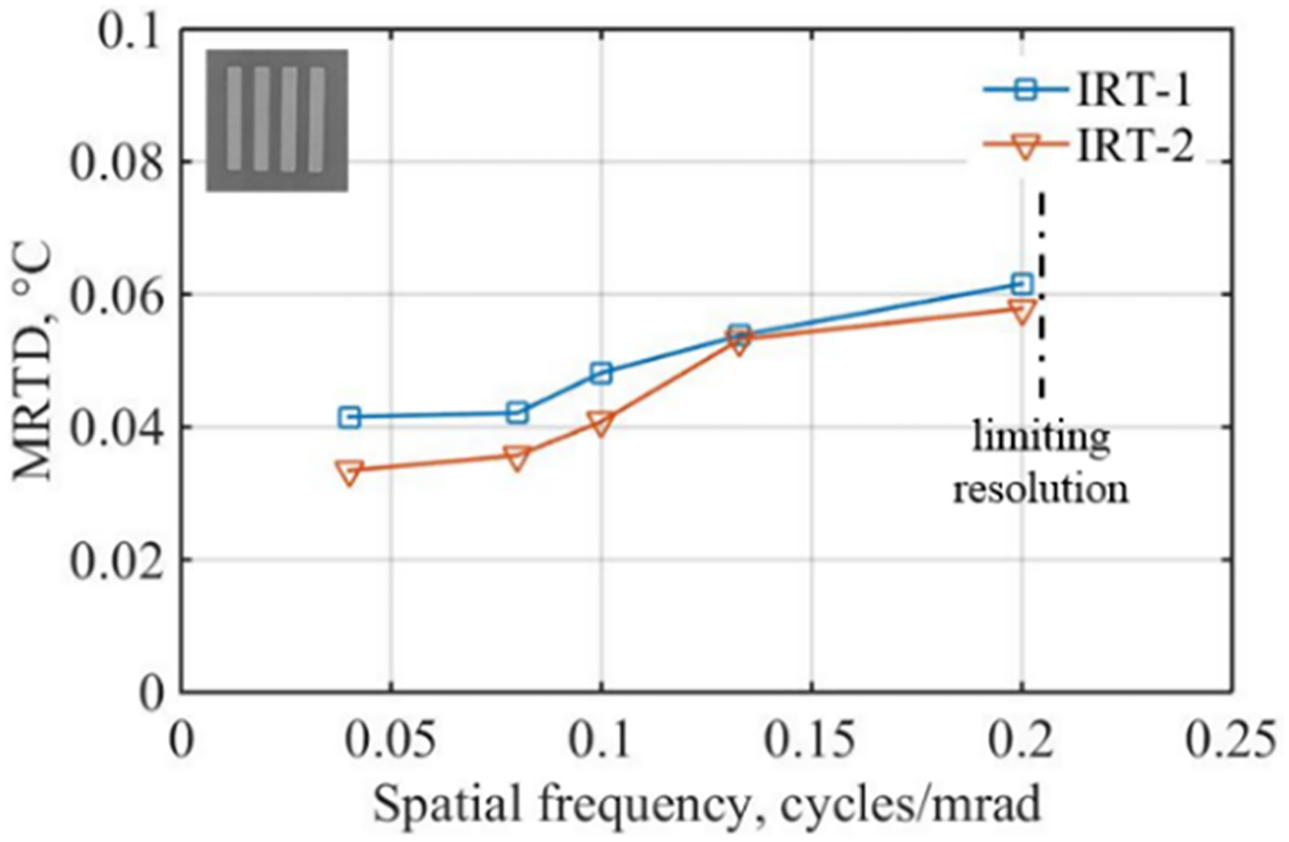
Effects of spatial frequency on MRTD. The conjugate bars were at ambient temperature and positioned in the vertical direction at 0.8 m. Reprinted from [39] under a CC BY license, with permission from SPIE Publications, original copyright [2017].

Results of MRTD measurements with target bars positioned in the vertical direction are presented in Fig 6 for both IRTs. As expected, the value of MRTD increases gradually with spatial frequency. The values at 0.2 cycles/mrad were 50% and 70% higher than the values at 0.04 cycles/mrad for IRT-1 and IRT-2, respectively. That is because (1) the contrast of smaller bars is reduced by the imager (a property of the imager that can be described with modulation transfer function), and (2) a larger temperature difference is needed for the observers to resolve a smaller bar set (a property of human eyes that can be described with contrast sensitivity function [55, 56]). The MRTD of IRT-2 was always smaller than that of IRT-1 probably because of its larger pixel number, which indicated the sensitivity of IRT-2 is better than that of IRT-1 –except for spatial frequency of 0.13 cycles/mrad, where both IRTs showed similar MRTD level. In general, MRTD values were bounded between 0.03°C to 0.06°C and therefore satisfied the standard requirement (less than 0.1°C—clause 201.101.5).

**Fig 7:**
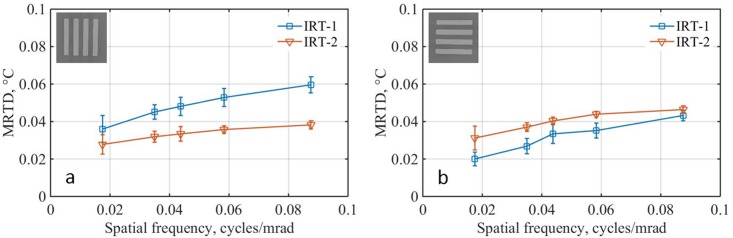
Effects of target temperature on MRTD. (a) vertical and (b) horizontal bars at 0.35 m. MRTD values averaged over five different target temperatures between 31°C and 39°C. Error bars show the data SD. Insets show sample high contrast thermographs acquired from the target at spatial frequencies of 0.018 cycles/mrad.

Results of the MRTD experiment inside the temperature chamber indicated that IRT thermal sensitivity is less affected by the target absolute temperature ranging from 31 to 39 °C. This is likely because of the highly linear response of the IRTs in the narrow temperature range of interest (Fig 9). The change in MRTD values due to the change in target absolute temperatures was minimal for all the tested spatial frequencies, as indicated by the small error bars in Fig 7. This change was measured to be 12% for IRT-1 (13% and 11% with the bars in horizontal and vertical directions, respectively) and 9% for IRT-2 (8% and 10% with the bars in horizontal and vertical directions, respectively). Since human face temperature usually ranges from 34 °C to 36 °C, we recommend the bar target temperature for MRTD measuring is set within this range.

Mean MRTD values measured at five different target temperatures as a function of spatial frequencies are shown in Fig 7 for vertical and horizontal target bars. IRT-1 showed higher MRTD values than IRT-2 for vertical bars. This is in part due to the vertical striping noise seen in IRT-1 (Fig 3a), which makes it more challenging for observers to visually resolve low contrast vertical bars. On the other hand, the MRTD values of IRT-1 are lower than those of IRT-2 for horizontal bars. For all cameras in both the vertical and horizontal directions, the MRTD values increase monotonically with spatial frequency. We propose to define the *u*_*MRTD*_ as the difference of MRTD values between the highest and lowest target frequencies with the similar rationale as the definition of *u*_*D*_.

For the same camera under the same measuring condition, the MRTD values in the horizontal and vertical directions might be different because of optical aberrations, the aspect ratio of sensor pixels, interline transfer architecture of the sensor, interlaced-to-progressive video conversion, *etc*. Therefore, the MRTD values in both vertical and horizontal directions should be measured and the uncertainty in both directions (*u*_*MRTD*_*V*_ for vertical direction and *u*_*MRTD*_*H*_ for horizontal direction) should be calculated. It should be noticed that vertical and horizontal bars measure MRTD in horizontal and vertical directions respectively. The MRTD uncertainty (*u*_*MRTD*_) should be the average of *u*_*MRTD*_*V*_ and *u*_*MRTD*_*H*_.

**Fig 8:**
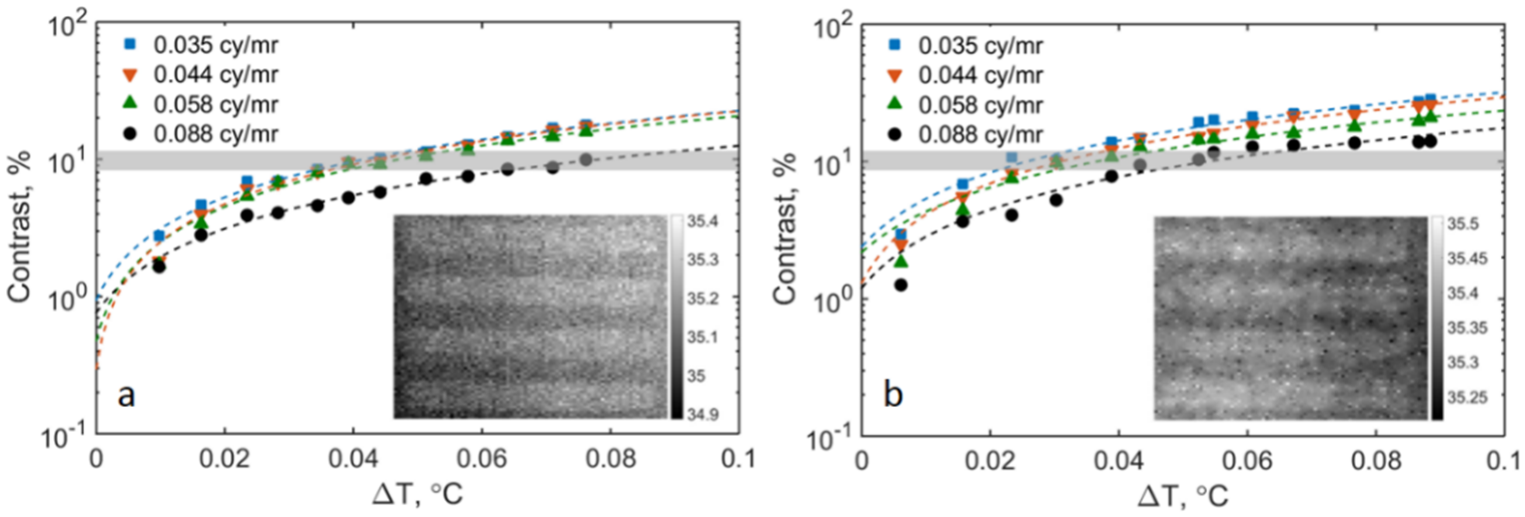
Contrast versus ΔT for (a) IRT-1 and (b) IRT-2. Shaded horizontal bar represents the lowest visible contrast of 10%. The 4-bar target (conjugate bars) maintained at 35°C and positioned in the horizontal direction at distance of 0.35m. Insets show sample thermograms at MRTD threshold for spatial frequency of 0.035 cycles/mrad.

Quantitative contrast measurements of the 4-bar target are presented in Fig 8 (results with the bars in vertical direction are not shown here). Based on the bar target images provided to the observers for MRTD evaluation (Section 2.3.3), the mean contrast corresponding to the visually resolvable bars (oriented in both horizontal and vertical directions) across different spatial frequencies is around 10% for IRT-1 and IRT-2 (the horizontal gray bars in Fig 8). If we define the minimum visible contrast as 10% [57], MRTD values could be estimated from the corresponding ΔT directly without using observers, which will significantly simplify the MRTD measurement.

### 3.4. Radiometric temperature laboratory accuracy

The radiometric temperature laboratory accuracy should satisfy Eq 6 over the range of at least 34°C to 39°C at no less than five CS temperature points (clause 201.101.2.2). At each CS temperature point, the values of |*T*_*ST*_ − *T*_*CS*_| and |*u*| should comply with Eq 6. The value of *u* is based on the values of *u*_*CS*_, *u*_*ETRS*_, *u*_*D*_, *u*_*S*_, *u*_*U*_, and *u*_*MRTD*_. Since we did not have *u*_*CS*_ and *u*_*ETRS*_ values at different temperatures and the main purpose of this paper is to develop characterization methods instead of evaluating devices, we assume the values of *u*_*CS*_, *u*_*ETRS*_, *u*_*D*_, *u*_*S*_, *u*_*U*_, and *u*_*MRTD*_ are the same within this temperature range. For complete evaluation of a device, these parameters should be measured at no less than 5 CS temperature points.

**Fig 9:**
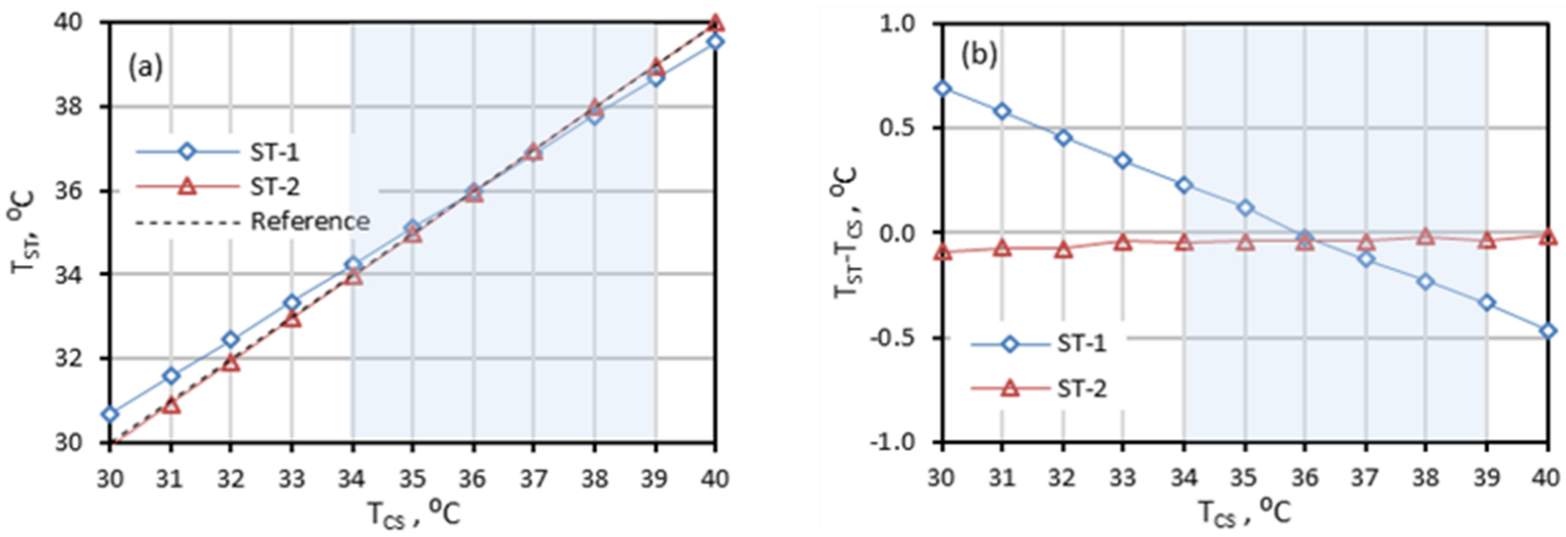
Laboratory accuracy. (a) response graph: T_ST_ versus T_CS_ (small error bars are not apparent in the graph), and (b) Bland-Altman graph: offset error versus T_CS_. Shaded area represents the required evaluation range.

Graphs of measured temperatures by the STs (*T*_*ST*_) and their corresponding offset errors (*T*_*ST*_ − *T*_*CS*_) (Bland-Altman plots [58, 59]) at various reference temperatures (*T*_*CS*_, the setting temperature of the CS) are given in Fig 9. Response curves for both STs are linear and each symbol represents the mean temperature calculated for a center region covering about 80% of the entire blackbody face. In Fig 9a, error bars from three repeated measurements are much smaller than the symbols and therefore not visible. Over the temperature range of 34°C to 39°C (shaded area in Fig 9), ST-1 showed a linear offset error that monotonically decreased from +0.23°C to -0.33°C, whereas ST-2 had a smaller offset error changing from -0.09°C to -0.01°C (Fig 9b). Since (*T*_*ST*_ − *T*_*CS*_) has a linear relationship with *T*_*CS*_, the largest value of |*T*_*ST*_ − *T*_*CS*_| over the range of 34°C to 39°C (in this case, 0.33°C for ST-1 and 0.09°C for ST-2) was applied in Eq 6.

To calculate the combined standard uncertainty values for ST-1 and ST-2, the values of *u*_*CS*_, *u*_*S*_, *u*_*D*_, *u*_*U*_, *u*_*MRTD*_, and *u*_*ETRS*_ are needed. The values of *u*_*CS*_ and *u*_*ETRS*_ were provided by the blackbody manufacturer (Table 2). For accurate evaluation, the blackbodies should be independently calibrated and traceable. The values of *u*_*S*_ and *u*_*D*_ were based on the methods and definitions in Section 2.3.1. The values of *u*_*U*_ were based on the all-pixels-SD method in Section 2.3.2. The values of *u*_*MRTD*_ were based on the methods in Section 3.3 and were the average values of *u*_*MRTD*_*V*_ and *u*_*MRTD*_*H*_. Since we only have *u*_*MRTD*_*H*_ data at working distance of 0.8 m (Fig 6) and the *u*_*MRTD*_*V*_ and *u*_*MRTD*_*H*_ data are close at other distance (Fig 7), we used *u*_*MRTD*_*H*_ as *u*_*MRTD*_ directly just for demonstration purpose. For an accurate evaluation, both the *u*_*MRTD*_*V*_ and *u*_*MRTD*_*H*_ should be measured. Table 4 shows all the standard uncertainty, combined standard uncertainty, and |*T*_*ST*_ − *T*_*CS*_| values for calculation of the radiometric temperature laboratory accuracy.

**Table 4:** List of standard uncertainties, combined standard uncertainties, and |*T*_*ST*_ − *T*_*CS*_| values.

	Standard uncertainties, °C						Combined standard uncertainty, °C	Offset errors, °C
ST	*u*_*D*_	*u*_*S*_	*u*_*U*_	*u*_*MRTD*_	*u*_*ETRS*_	*u*_*CS*_	|*u*|	|*T*_*ST*_ − *T*_*CS*_|
ST-1	0.03	0.02	0.11	0.02	0.04	0.04	0.13	0.33
ST-2	0.08	0.02	0.05	0.02	0.04	0.04	0.11	0.09

Note: *u*_*ETRS*_ and *u*_*CS*_ are from Table 2. The offset errors only show the largest value within temperature range of 34–39 °C.

The sum of |*T*_*ST*_ − *T*_*CS*_| and |*u*| is 0.46 °C for ST-1 and 0.20 °C for ST-2, indicating both ST-1 and ST-2 satisfy the laboratory accuracy requirement (Eq 6) over the 34°C to 39°C range. The main differences between ST-1 and ST-2 are the uniformity and offset error. The *u*_*U*_ values for ST-1 and ST-2 are 0.11 °C and 0.05 °C respectively. Since the image sensor in ST-2 has more pixels than the sensor in ST-1, only the region with the best uniformity in ST-2 was defined as the WTP region. Therefore, ST-2 has better uniformity results. The CS uncertainty value can significantly affect the offset errors. If the total system uncertainty of the CS in Table 2 is not accurate, the offset errors in Table 4 and thus the laboratory accuracy values of ST-1 and ST-2 will be different.

## 4. Discussion

This paper has implemented and evaluated essential performance test methods recommended for fever screening thermographs in IEC 80601-2-59 [30]. Modifications to future implementation or revisions of this standard have been suggested. Our performance evaluation of two moderately priced IRTs in a controlled laboratory environment found that both devices—as a part of a ST system—may meet the stated performance requirements if well-established experimental test methods are implemented except for the uniformity of the WTP. A summary of our findings for the essential performance of the two ST systems under study are listed in Table 5.

**Table 5:** Essential performance requirements for STs.

Performance factors	Requirements [30]	Our results	Comments
Stability	Clause 201.101.4: Stability should be <0.1°C	ST-1 and ST-2 satisfied the requirement.	None of the IRTs alone met the requirement. Offset temperature compensation using an ETRS to convert an IRT to a ST is essential.
Drift	Clause 201.101.4: Drift measurement taken over 14 days or the system calibration interval whichever is longer. Drift should be <0.1°C	ST-1 and ST-2 satisfied the requirement.	None of the IRTs alone met the requirement. Offset temperature compensation using an ETRS to convert an IRT to a ST is essential. We evaluated drift over a two-week period. A measurement interval longer than one month is recommended.
Uniformity	Clause 201.101.6: Uniformity evaluation based on measurements at random locations. Uniformity should be <0.2°C	ST-1 and ST-2 failed to satisfy the requirement based on the IEC-29-pixels method.	Offset temperature compensation using ETRS is essential for the IEC-29-pixels method. A less burdensome approach based on a single shot of a uniform BB can be a better solution. The imager should be focused at the working distance while evaluating the uniformity. Only IRT-2 satisfied the requirement based on the modified-29-pixels method. Both IRTs failed when the all-pixels method was used. The uniformity values based on the maximum difference varied significantly between repeated measurements and increased with the number of selected pixels. The SD-based all-pixels-SD method might be an alternative uniformity measure.
MRTD	Clause 201.101.5: A subjective MRTD evaluation method is suggested in ASTM E1213-14. MRTD should be < 0.1°C	ST-1 and ST-2 satisfied the requirements.	An alternative contrast-based objective method was proposed and examined. The direction and spatial frequency of etched bars could affect the MRTD results.
Laboratory accuracy	Clause 201.101.2.2: |*T*_*ST*_ − *T*_*CS*_ | + |*u*| ≤ 0.5	ST-1 and ST-2 satisfied the requirements within their WTPs.	None of the IRTs alone met the accuracy requirement. Offset temperature compensation using an ETRS is essential.

Measurements of stability and drift showed that both STs met the requirements specified in the standards. The temperature information collected from the ETRS were essential to increase stability and decrease drift of a ST. In general, combined stability and drift was improved by 80% and 95% for ST-1 and ST-2 than IRT-1 and IRT-2, respectively. These findings also provide quantitative support for the use of an ETRS during screening measurements, if necessary, as noted in the standard. For drift analysis, the standard requires that the experiment should be repeated every day for the device’s calibration interval or two weeks, whichever is longer. We measured the device drift over two weeks since our focus is on the evaluation methods instead of the device evaluation and we assume the ETRS has high stability and low drift and has been calibrated per the calibration interval. From the experience of thermal camera drift measurements at National Physical Laboratory, drift caused by lens and/or detector changes can only truly be determined over a period longer than one month. The standard requires data acquisition every 5–15 seconds for 8 hours each day for stability and drift measurement, which will accumulate a huge amount of data for drift measurement. We evaluated the stability and drift based on longer data acquisition intervals of 30, 60, 120 and 300 seconds, and got similar results for different intervals. Therefore, data acquisition every 5 minutes might be sufficient for stability and drift measurement.

Different uniformity evaluation approaches were implemented and compared. Multiple types of spatial artifacts were observed, including striping, vignetting, and narcissus artifacts. Therefore, for each individual IRT, spatial artifacts may need to be detected, analyzed and mitigated accordingly. While the focal length of IRT-1 didn’t significantly affect the uniformity, it did cause lens artifacts for IRT-2 (Fig 3c and 3d). Therefore, uniformity testing needs to be performed by focusing at the working distance but placing the CS at a shorter distance so that the CS is defocused to reduce its non-uniform effects on uniformity evaluation. Both IR cameras failed the uniformity tests following the standard (IEC-29-pixels method). This method should be further evaluated to see whether the threshold value is too tight.

The IEC-29-pixels method assumes that the imager is stable, and the CS is stable and uniform for the test period, which might not be true based on our stability data. The modified-29-pixels, all-pixels and all-pixels-SD approaches assume that the CS is uniform and promote this assumption by putting the CS at a defocused location. These methods are less burdensome than the IEC-29-pixels method since only one frame is needed and the CS doesn’t need to be moved around. Furthermore, no offset compensation is necessary for these methods since all the pixels are from the same frame, thus no stability problem exists. On the other hand, offset compensation with an ETRS is necessary for the IEC-29-pixels method since multiple frames are used.

When the uniformity is defined based on the maximum difference between pixel values, the evaluation results varied significantly between repeated processes, especially when the number of the sampled pixels was small (*e*.*g*., 29 pixels). The probability of obtaining a high uniformity value (indicating bad uniformity) increases with the number of sampled pixels. The all-pixels method showed the largest uniformity values since it considers the entire thermogram. On the other hand, our data have shown that SD based on a statistical analysis can be an alternative measure for uniformity evaluation and the all-pixels-SD method may be a choice for uniformity evaluation. The uniformity criterion of 0.2 °C based on the IEC-29-pixels method can be mirrored to a criterion of 0.05 °C based on the all-pixels-SD method. Considering the tested devices could barely meet these criteria, these criteria might be too high. A criterion around 0.1°C (Fig 4b) based on the all-pixels-SD method might be considered.

The MRTD results of this study indicate that the IRTs used were sensitive enough to resolve small temperature differences in vertical and horizontal directions and satisfied the standard requirement of less than 0.1°C. Our study shows that the orientation and spatial frequency of 4-bar targets affects MRTD results, which prior standards [30, 50] do not mention. We suggest measuring MRTD in both horizontal and vertical directions, at different spatial frequencies, and at different locations within the WTP (*e*.*g*., at the center and four corners of the WTP). The MRTD values in both directions and at different locations should be averaged. The technique recommended in the standards is subjective and time-consuming. The objective test method described in this paper could streamline the procedure and improve consistency. We suggest to average frames captured in 0.2 seconds and calculate the contrast level of the averaged frame to simulate the human eye/brain time integration system. Our data show that 10% contrast can be defined as the minimum recognizable contrast level. Therefore, the minimum temperature difference of a bar group whose image contrast is 10% can be defined as the MRTD.

The spatial frequencies of test targets have significant effects on MRTD measurement and *u*_*MRTD*_ calculation. In this paper, we defined *u*_*MRTD*_ as the difference of MRTD values between the highest and lowest target frequencies. Obviously, the *u*_*MRTD*_ values will be larger for a wider spatial frequency range (Fig 6). Therefore, defining a reasonable spatial frequency range is essential. The highest frequency a camera can detect is limited by both the optics (cutoff frequency) and the sensor pixel size (Nyquist frequency). Therefore, the highest spatial frequency for *u*_*MRTD*_ measurement should be the cutoff frequency or the Nyquist frequency whichever is smaller. The lowest spatial frequency for *u*_*MRTD*_ measurement should be a frequency that below which the MRTD value will not change.

Results indicated that the radiometric temperature laboratory accuracy of a ST can be affected by factors including stability, drift, MRTD, uniformity and the quality of the ETRS and CS. To optimize ST accuracy, an initial thermal stabilization time was always considered prior to each test. While the laboratory accuracy of both ST-1 and ST-2 satisfied the standard threshold, IRTs alone did not meet the standard accuracy requirements. The WTP of ST-2 was only a sensor region with the best uniformity.

A high-quality blackbody working as a CS or an ETRS is essential for performance testing of a ST. The evaluation data in this paper were based on the assumption that the blackbodies parameters in Table 2 are accurate. From experience of calibrating blackbodies at the National Physical Laboratory, the uncertainty values of an extended area blackbody are typically larger than the values in Table 2. Therebefore, the blackbodies should be independently calibrated for accurate evaluation. The standard requires the emissivity of the CS should be at least 0.998. However, such CS is difficult to find. Since the CS emissivity can be compensated based on the Stefan-Boltzmann formula, a CS with emissivity around 0.98 should be sufficient. The standard recommends the size of ETRS active area to be less than 10% of the face during fever screening. However, we showed that an ETRS with larger size (in our case: 15–20%) is also acceptable if it does not negatively affect the results.

The main purpose of this paper is to evaluate, demonstrate and improve the test methods for different IR performance characteristics, not to evaluate devices. Therefore, we only evaluated the standard uncertainty values of different characteristics once for a given device under a specific environment. To completely evaluate the performance of a given model of thermographs, several devices of the same model should be evaluated by multiple engineers in different laboratories to elucidate the effects of device-to-device variations, human factors, and test environments, which is beyond the scope of this paper.

## 5. Conclusion

Our research into performance evaluation of the IR thermography systems has provided significant insights toward the design of least burdensome standardized test methods. It is our intent that these insights can be used to build on prior excellent work in IRT standards to help advance screening thermographs as an accurate, non-invasive clinical tool with practical application for mitigating the severity of future pandemic disease outbreaks. The main purpose of this paper was to evaluate and modify test methods for a specific device under a designed application environment. The test data for the devices can be affected by many factors (*e*.*g*., location and size of WTP can significantly affect uniformity results; blackbody uncertainty can affect laboratory accuracy).
